# Thromboelastography in Microsurgical Reconstruction: A Systematic Review

**DOI:** 10.1016/j.jpra.2021.12.005

**Published:** 2022-01-11

**Authors:** M. Thakkar, A. Rose, B. Bednarz

**Affiliations:** 1 Department of Plastic and Reconstructive Surgery, St Mary's Hospital, Praed Street, London, W2 1NY; 2 Department of Plastic and Reconstructive Surgery, Glasgow Royal Infirmary, 84 Castle Street, Glasgow, G4 0SF; 3 Department of Plastic and Reconstructive Surgery, Southmead Hospital, Southmead Road BS10 5NB

**Keywords:** TEG, ROTEM, Thromboelastography, Thromboelastometry, Free flaps, Free tissue transfer, Microsurgical reconstruction

## Abstract

The aim of this review was to identify studies that used thromboelastography (TEG) or rotational thromboelastometry (ROTEM) in microsurgical free flap reconstruction and analyse whether it is a useful adjunct at predicting and identifying thrombotic complications.

A search was conducted using the MEDLINE database using the keywords “thromboelastogram”, “TEG”, “thromboelastography”, “free flaps” and “free tissue transfer” using a two-component search with the Boolean operators “OR” and “AND”.

Eight studies were retrieved using the search criteria. Seven studies met the inclusion criteria, and a further study was found citing several articles from the initial search. Combined, there were 528 patients who underwent 600 free flaps. A total of 10.3% (62) arterial and venous thromboses were reported in the studies, and the combined flap failure rate was 5.2% (26).

A total of 67% (4/6) of the studies supported the use of TEG as a predictive tool to detect thromboses, including three retrospective case series and one prospective cohort, which were all statistically significant.

There is low-quality evidence (level IV) that a pre-operative TEG and functional fibrinogen to platelet ratio of ≥42 can identify patients at risk of adverse post-operative thrombotic events following free flap surgery; however, further validation is required. Higher quality, standardised prospective or randomised control trials are required to further evaluate the predictive value of TEG. As a pre-operative screening tool, TEG can help to detect pathological changes in coagulation, aid in the transfusion of blood products, target anticoagulation therapy and predict possible adverse events aiding to further reduce patient morbidity.

## Introduction

Microsurgical reconstruction has become the gold standard choice for the reconstruction of complex defects. Technical advancements and improved flap monitoring techniques have led to success rates of >95%.^1^ Despite the high success rates, vascular complications such as arterial or venous anastomotic thromboses are still a threat and can jeopardise the outcomes of such reconstructions leading to additional surgery, morbidity and flap failure. Flap complications and flap failure can occur due to a multitude of factors that often overlap and compound, including patient comorbidities, zone of trauma, surgical factors (technical errors) and anaesthetic related factors (hypoxia, hypotension and acidosis). Ultimately, flap failure is usually as a result of pedicle thrombosis due to local, systemic (coagulopathy) or technical factors such as pedicle kinking or intimal injury.^2^ Having a reliable method to identify patients at high risk of thrombosis during the pre-operative, peri-operative and post-operative period could help to predict, risk stratify and prevent such disastrous consequences from occurring helping to further improve success rates.

Thromboelastography (TEG®, Haemonetics, Boston, MA, USA) is a quick dynamic point-of-care testing method that measures the efficiency of blood coagulation. It is a viscoelastic haemostatic assay that depends on the interaction between the plasmatic coagulation system, platelet function, fibrinolysis and other factors to measure the speed and strength of clot formation (aggregation, clot strengthening, fibrin cross-linking and fibrinolysis).^3^ Standard laboratory coagulation tests such as prothrombin time (PT) and activated partial thromboplastin time (APTT) measure coagulation factor function; however, they cannot measure haemostasis as a whole. It is widely used to help guide transfusion management in settings such as trauma, critical care and cardiac surgery.^4^ Rotational thromboelastometry (ROTEM®, Pentapharm GmbH, Munich, Germany) is a different type of commercially available viscoelastic assay that has different diagnostic nomenclature for identical parameters. In simple terms, the sensor shaft oscillates instead of the cup in ROTEM.

The aim of this review was to identify studies that used TEG or rotational thromboelastometry in microsurgical free flap reconstruction and analyse whether it is a useful adjunct at predicting and identifying thrombotic complications.

## Methods

The PRISMA guideline on reporting systematic reviews was adhered to as much as possible.^5^ A search was carried out using the PubMed database. The keywords “thromboelastogram”, “TEG”, “thromboelastography”, “free flaps” and “free tissue transfer” were used in a two-component search using the Boolean operators “OR” and “AND”. Abstracts extracted were reviewed by the authors to further narrow down the search, and eligibility was determined using the following inclusion and exclusion criteria. Inclusion criteria included any series of patients in which TEG or ROTEM was used in the context of microsurgical reconstruction. Exclusion criteria included non-English language publications and usage in non-microsurgical reconstruction.

Data extracted from the studies included country, year, study type, population, outcomes, mean age, gender distribution, flap types, flap indication, flap complications, flap failure rate, study aim, study protocol and key results. Levels of evidence were assigned to each study according to the Oxford levels of evidence.^6^

Data were analysed subjectively taking into account key outcomes due to the lack of homogeneity between studies in terms of study protocol, type of assay used and variables measured.

## Results

The database search yielded eight results. After evaluation, seven studies met the inclusion criteria. A further study was included that was discovered citing several articles from the initial search. There were no randomised control trials available. Two studies were prospective cohorts, four studies were retrospective case series, and two were case reports.

Demographic data extracted from the studies are shown in Tables 1 and 2 below. Aims, measurement protocols and key outcomes are shown in Table 3.

**Table 1:** Population, overall outcome, study type and attributed level of evidence

Study	Country	Population	Outcome	Study type	Level of evidence^6^
Parker et al. 2012^8^	UK	29 patients undergoing free tissue transfer for head and neck pathology	A functional fibrinogen to platelet ratio above 42% as measured by TEG may be useful in identifying patients likely to develop thrombotic complication	Retrospective case series	4
Murphy et al. 2013^9^	UK	Single patient with myelodysplastic syndrome undergoing extensive periorbital reconstruction	The use of TEG allows for targeted clotting supplementation which may reduce over-correction of platelet and/or clotting factor deficiencies and reduce free flap complications	Case report	5
Kolbenschlag et al. 2014^10^	Germany	181 consecutive patients undergoing free flap surgery	ROTEM seems to be able to identify patients that are prone to thrombotic complications and might be used as a screening tool	Retrospective case series	4
Wikner et al. 2015^11^	Germany	35 patients undergoing free flap surgery	The utilisation of thromboelastometry allows for assessment of the anticoagulation needs of individual patients undergoing free flap surgery	Prospective cohort	3
Zavlin et al. 2018^12^	USA	2 patients with factor V Leiden undergoing DIEP reconstruction	TEG was used in the first 72 hours to monitor patient's hypercoagulability and prevent flap thrombosis	Case report	5
Zavlin et al. 2018^13^	USA	100 consecutive patients undergoing abdominal free flap reconstruction (172 flaps)	TEG is a useful adjunct for monitoring coagulation status in microsurgical breast reconstruction	Retrospective case series	4
Ekin et al. 2019^14^	Turkey	77 patients undergoing free flap reconstruction	There was no significant relationship between pre-operative and post-operative TEG and flap complications and loss	Retrospective case series	4
Vanags et al. 2020^7^	Latvia	103 consecutive adult patients with traumatic injuries undergoing free flap surgery	In the late surgery group, thromboelastometry supports the detection of hypercoagulability and predicts free flap thrombosis risk	Prospective cohort	3

**Table 2:** Mean age, gender distribution, flap types, flap indications, complications and flap failure rate reported in the studies

Study	Mean age	Gender	Flap types	Trauma/malignancy/ infection	Flap complications	Flap failure rate
Parker et al. 2012^8^	58	17M, 12F	Radial forearm, fibula, LD, groin, scapular	26 malignancy, 3 benign	Arterial thromboses 2, venous thromboses 3	13.8% (4)
Murphy et al. 2013^9^	68	1M	ALT	Infection	Nil	nil
Kolbenschlag et al. 2014^10^	50.2	108M, 73F	ALT, LD, DIEP, parascapular, gracilis, fibula, lateral arm, S-GAP	108 trauma, 45 malignancy, 12 infection, 9 burns, 7 chronic ulcers	28 thromboses, 15 venous, 6 arterial, 7 arterial and venous	7.7% (14)
Wikner et al. 2015^11^	61.8	20M, 15F	Radial, fibula, ALT, scapular, ulnar	27 malignancy, 15 chronic skin ulcers	7 bleeding events, 5 thrombotic events	8.6% (3)
Zavlin et al. 2018^12^	48.5	2F	DIEP	Abdominal breast reconstruction	Nil	Nil
Zavlin et al. 2018^13^	48.2	100F	DIEP, SIEA	Abdominal breast reconstruction	1 arterial thrombosis, 2 venous thromboses, 1.7% bleeding, 4.7% wound infection	1.2% (2)
Ekin et al. 2019^14^	49.3	40M, 37F	DIEP, fibula, ALT, radial forearm, LD	Not stated (elective free flaps)	5.2% partial necrosis, 6.5% thromboses (5), 10.4% hematoma, 7.8% wound dehiscence, (1.3%) seroma	3.9% (3)
Vanags et al. 2020^7^	40.4	90M, 13F	Scapular, parascapular, ALT, LD, Fibula	Traumatic injuries	16 thromboses within first 24 hours	Not reported

**Table 3:** Aims, type of assay, measurement protocol and key results extracted from each study (case reports excluded)

Study	Aim	TEG or ROTEM	Protocol	Key results
Parker et al. 2012^8^	To determine if functional fibrinogen to platelet ratio using TEG could pre-operatively identify patients at risk of developing thrombotic complications	TEG	TEG analysis and calculation of functional fibrinogen to platelet ratio at induction of anaesthesia	• The mean functional fibrinogen to platelet ratio was significantly higher in the surgery group compared with healthy volunteers • Of the 29 patients studied, 31% (n=9) had some form of thrombotic event, with all but one patient having a functional fibrinogen to platelet ratio >42% (mean 47% ±7%)
Kolbenschlag et al. 2014^10^	To assess the diagnostic value of rotational thromboelastometry (ROTEM) regarding thrombotic complications in reconstructive microsurgery	ROTEM	Pre-operative day, standard laboratory work-up, including a coagulation screening and RTE measurement	• Pre-operatively, 36.5% of patients had a hypercoagulable ROTEM (higher than physiological ROTEM values) • A total of 28 primary thromboses of the microvascular pedicle occurred, 11 of these occurred in-patients with a hypercoagulable state • Both a hypercoagulable ROTEM assay and a functional fibrinogen to platelet ratio (FPR) of >43 were significant predictors of thrombotic flap loss when performing multivariate binary logistic regression, co-factoring for age, sex and comorbidities (p=0.036 and 0.003, respectively)
Wikner et al. 2015^11^	To evaluate the systematic use of thromboelastometry in the peri-operative course in cranio-maxillofacial free-flap patients in comparison with standard testing and prove its value prospectively, outlining alterations for clinical applicability in hospitals using antithrombotic agents	ROTEM	Blood samples were obtained at three defined time points: 1) Pre-operative coagulation status: beginning of surgical procedure 2) Establishment of anastomosis 3) 24 h after establishment of anastomosis At each point, ROTEM with special regard to clotting times for the intrinsic and extrinsic paths of coagulation was immediately performed Laboratory parameters comprised of prothrombin time (PT), partial thromboplastin time (pTT), thrombin time (TT) and platelet count (PC). Clinical routine parameters also included a full blood count, markers of inflammation and electrolytes	• Neither standard testing nor ROTEM were capable of predicting adverse events such as thrombosis, bleeding or flap loss (p > 0.05)
Zavlin et al. 2018^13^	To investigate if there is a value of TEG in identifying patients at risk for microvascular thrombosis or bleeding	TEG	TEG analysis was carried out at intervals, including pre-operative (baseline), intra-operatively, after surgery on post-operative day 1 (POD1) and 2 (POD2) Basic coagulation studies, such as thrombocyte count, prothrombin time (PT) and activated partial thromboplastin time (aPTT) were measured at baseline, intraoperatively and POD2	• Classical coagulation studies, such as thrombocyte levels, aPTT and PT failed to identify patients with thrombosis • Patients with thromboses had significantly larger TEG-G boosts after surgery compared with the control cohort (p=0.049) • Sharp increases of the TEG-G value right after surgery could therefore be a predictor of flap failure
Ekin et al. 2019^14^	To evaluate the coagulation status of elective free flap reconstructive surgery patients with conventional tests and TEG and to investigate the effect of the coagulation status on flap complications and flap success The second aim was to identify other comorbidities that may lead to flap complications and flap loss	TEG	Pre-operative and post-operative blood tests (haemogram and standard coagulation tests) and TEG results were recorded	• Laboratory test results revealed no statistical correlation between flap complications and flap loss with pre-operative and post-operative TEG
Vanags et al. 2020^7^	To assess the role of ROTEM as a means of early identification of hypercoagulability in trauma patients prone to develop free flap thrombosis Thrombotic risk factors and duration of surgery were evaluated as secondary outcomes	ROTEM	36 patients with recent trauma underwent surgery within 30 days (ES group) were compared with 67 trauma patients who underwent surgery later than 30 days (late surgery, LS group) after the injury ROTEM was performed before surgery Functional fibrinogen to platelet ratio (FPR) ≥ 42 was selected as the main hypercoagulability index	• Six patients (16.7%) in the ES group and 10 (14.9%) in the LS group had free flap thrombosis (not significant (NS)) • Hypercoagulability occurred more frequently in the ES group (44.4%) than in the LS group (11.9%; p < 0.001), it was not associated with higher free flap thrombosis rate • In LS patients, hypercoagulability increased the odds of free flap thrombosis (OR 8.83, CI 1.74–44.76; p = 0.009) • A positive correlation was found between FPR ≥ 42 and free flap thrombosis rate (r = 0.362; p = 0.003)

Overall, there were a total of 528 patients who underwent 600 free flaps. The average age was 53.1. In terms of gender, 276 patients were male and 252 female. A total of 62 (10.3%, 62/600) arterial and venous thromboses were reported in the studies and the combined flap failure rate was 5.2% (26/497, *flap failure rate not reported by Vanags et al.^7^).

In terms of supporting the ability of TEG as a predictive tool to detect thromboses, 67% (4/6) of the studies supported it, including three retrospective case series and one prospective cohort which were all statistically significant. This is shown in Table 4.

**Table 4:** Outcomes of studies supporting or opposing the predictive ability of thromboelastoraphy to predict adverse thrombotic events

Predicts adverse events	Do not predict adverse events
Parker et al. 2012^8^⁎	Wikner et al. 2015^11^ (prospective cohort)
Kolbenschlag et al. 2014^10^⁎	Ekin et al. 2019^14^
Zavlin et al. 2018^13^⁎	
Vanags et al. 2020^7^⁎ (prospective cohort)	

⁎ denotes statistical significance P<0.05.

## Discussion

TEG is a dynamic point-of-care viscoelastic haemostatic assay that gives a real-time image of in vitro clot formation by taking into account not only the plasmatic components of coagulation but also the cellular elements and their interactions. It is able to produce a global reflection of the patients coagulatory state, including hypercoagulable and coagulopathic states. TEG is widely used in trauma, critical care and cardiac surgery. In fact, TEG analysis has been shown to be able to detect hypercoagulable states and predict thromboembolic events in trauma patients.^15^^,^^16^ It measures the physical properties of a clot via a pin suspended in a cup. As the elasticity and strength of the developing clot changes, it alters the rotation of the pin which in turn is converted into an electric signal which a computer uses to produce a graphical and numeric output. Each TEG run takes 30–60 min to complete depending on the device.

The R value is the reaction time and is the latency from the start to the initial fibrin formation at which the pin is 2 mm deviated and is dependent on clotting factors. K is the kinetics and is the time taken to achieve a clot strength of 20 mm amplitude. It is the amplification stage dependant on fibrinogen. The α angle represents the gradient between R and K and measures the speed at which fibrin build up and cross-linking takes place. TMA represents time to maximal amplitude. MA is maximal amplitude and represents the maximum size and strength of clot before fibrinolysis begins. A30 represents the amplitude at 30 min post-MA. CLT represents clot lysis time. The G value is a log-derivation of the MA tracing used in the study by Zavlin et al.^13^ As mentioned previously, ROTEM has different diagnostic nomenclature for identical TEG parameters, as shown in Table 5.

**Table 5:** The different diagnostic nomenclature for identical parameters between TEG and ROTEM

TEG	ROTEM
R value	Clotting time (CT)
K value and α angle	Clot formation time (CFT) and α angle
Maximal amplitude (MA)	Maximum clot firmness (MCF)
A30 or L30	Clot lysis

Parker et al.^8^ use a functional fibrinogen to platelet ratio (FPR) with a cut off of above 42% to define hypercoagulability. This was calculated using an additional TEG that removed the platelet component to measure the fibrinogen contribution to clot strength. Vanags et al.^7^ and Kolbenshlag et al.^10^ also use this ratio in their studies as a measure of hypercoagulability.

Pedicle thrombosis and flap failure are a devastating consequence of microvascular reconstruction for both patients and surgeons. Prudent monitoring of flaps in the post-operative period is currently the optimal method to identify pedicle thrombosis early leading interventions that allow potential flap salvage. There are multiple scoring systems that help to identify patients at risk of systemic venous thromboembolism;^17^ however, currently, there are none yet that identify patients at risk of pedicle thrombosis. TEG is such a screening tool that allows pre-operative screening of the patients coagulatory state with the ability to identify patients at high risk of pedicle thrombosis. These patients can also be targeted with interventions such as anticoagulatory therapy or more prudent invasive monitoring in order to further increase success rates. Dai et al.^18^ in their systematic review showed that the predictive accuracy of TEG for thromboembolic events is highly variable due to variations in the definition of hypercoagulability, TEG methodology, patient characteristics, reference standards and outcomes measured. It was not possible for them to carry out a meta-analysis and they called for more prospective studies. Interestingly, they state that maximal amplitude seems to be the best parameter to identify hypercoagulable states and to predict thromboembolic events.^18^ Zahr Eldeen et al. showed that in 823 adult patients undergoing liver transplantation, pre-operative TEG, in particular maximum amplitude (MA), was able to reliably identify the group of recipients at greater risk of developing early hepatic artery thrombosis.^19^

Parker et al.^8^ were the first to study the effect of TEG to predict thrombotic complications in microsurgery. Their preliminary study included 29 patients undergoing free flap reconstruction for head and neck pathologies. Although their sample size was small, they postulated that a functional fibrinogen to platelet (FPR) ratio of ≥42% has a sensitivity of 89% and specificity of 75% for predicting thrombotic events. This study was followed up by Kolbenschlag et al., who in their retrospective series of 118 patients reported that a hypercoagulable ROTEM or FPR of >43 placed patients at a significantly higher risk of thrombotic flap loss.^10^ Notably, Zavlin et al. in their series of 100 abdominal breast reconstructions did not find any link between pre-operative TEG measurements and thrombotic events.^13^ Baseline TEG values were used to administer unfractionated heparin according to an algorithm developed by the senior author and attributed to the low flap loss rate of 1.2%. Compared with control groups, they found that thrombotic cases had much steeper increases in TEG-G between surgery and post-operative day 2. In the most recent study, Vanags et al. conducted a prospective cohort study on patients with traumatic injuries scheduled for free flap surgery.^7^ Patients were divided into two groups undergoing early surgery < 30days or later surgery >30 days. Strikingly, hypercoagulability (FPR≥42) occurred more frequently in the early surgery group but was not associated with a higher free flap thrombosis rate compared with increased surgical time. In contrast, in the late surgery group, hypercoagulability was found to be a significant risk factor for free flap thrombosis.

In their prospective cohort of 35 patients undergoing free flaps for malignant tumours of the head and neck, Wikner et al. did not detect any predictive capability of thromboelastometry measurements for events such as pedicle thrombosis, bleeding or flap loss.^11^ Finally, Ekin et al. in their retrospective series of 77 patients could not detect any statistically significant correlation between TEG measurements pre and post-operatively.^14^

Mirroring the findings of Dai et al.^18^ it is clear that there are a few studies that are highly variable in terms of quality and methodology. Due to the differing study protocols, statistical analysis, definition of hypercoagulability, TEG measurements and outcome measures, it is not possible to conduct a meta-analysis to quantitatively compare studies. It is glaringly obvious from reviewing the studies that there are a wide variety of non-standardised anticoagulation regimens employed by surgeons such as the algorithm based on TEG measurements in the study by Zavlin et al.^13^ and no overall consensus amongst microsurgeons.^20^^,^^21^ The lack of a clear evidence-based anticoagulation protocols may confound the predictive value of TEG measurements in detecting adverse outcomes; however, the lack of microsurgical experience and bad technique can never be compensated by any regimen of antithrombotic therapy.^22^

Wang et al. in their review of 58 free flaps in hypercoagulable patients (diagnosed thrombophilia or previous thromboembolic events) had a thrombosis rate of 20.7%, 0% flap salvage rate for post-operative thrombosis and a flap loss rate of 15.5%.^23^ This indicates the high risk of adverse events in the hypercoagulable patient which TEG analysis may identify and allow intensified anticoagulatory therapy for high-risk individuals.

Indeed, flap success depends on a multitude of factors such as surgical technique, experience, patient co-morbidities, intraoperative anaesthesia management (such as maintaining haemodynamic stability) and careful post-operative monitoring.

**Figure 1:**
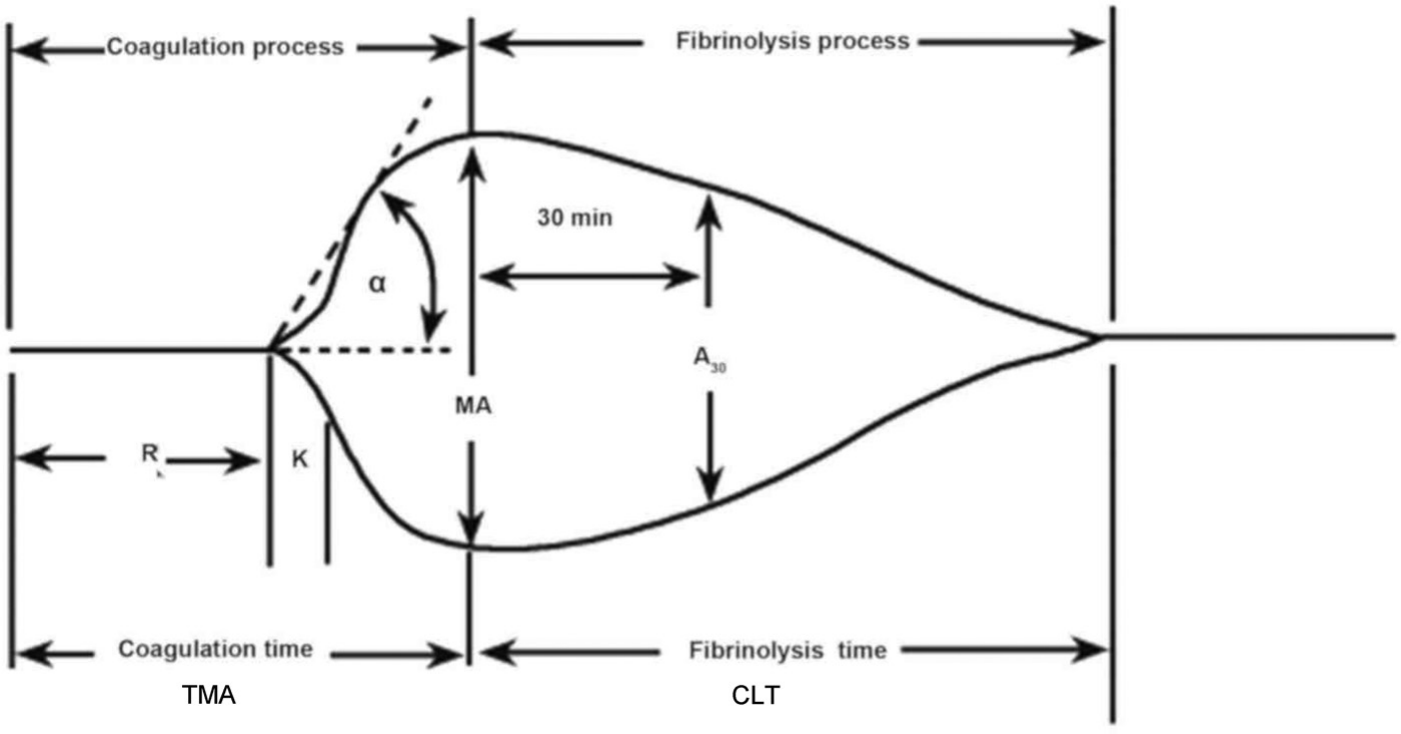
Normal TEG waveform

Limitations of this review include the search of a single database, lack of bias and qualitative assessment of each individual study and the low number of studies found Figure 1.

## Conclusion

Currently, the studies available are highly variable and difficult to standardise. There is low-quality evidence (level IV) that a pre-operative TEG and functional FPR of ≥42seems to identify patients at risk of adverse post-operative thrombotic events following free flap surgery and might be useful as a screening tool; however, further validation is required. Higher quality, standardised prospective or randomised control trials are required to further explore the predictive value of TEG. As a pre-operative screening tool, TEG can help to detect pathological changes in coagulation, aid in the transfusion of blood products, target anticoagulation therapy and predict possible adverse events aiding to further reduce patient morbidity.

## Declaration of Competing Interest

No funding to declare
